# A Fast-Fourier-Transform-Based Dynamic Likelihood Ratio Framework for Controlling False Positives in DNA Database Matching

**DOI:** 10.3390/genes17050499

**Published:** 2026-04-23

**Authors:** François-Xavier Laurent, Willem Burgers, Wim Wiegerinck, Cyril Gout, Susan Hitchin

**Affiliations:** 1 DNA Unit, International Criminal Police Organization—INTERPOL, 200 Quai Charles de Gaulle, 69006 Lyon, France; c.gout@interpol.int; 2 Bonaparte Team, SMART Research BV, Heyendaalseweg 135, 6525 AJ Nijmegen, The Netherlands; w.burgers@smart-research.nl (W.B.); w.wiegerinck@smart-research.nl (W.W.)

**Keywords:** DNA, DNA database, DNA matching, likelihood ratio, dynamic LR threshold, Fast Fourier Transform, Probability Mass Functions, Prüm DNA exchange, INTERPOL

## Abstract

**Background/Objectives**: Operational DNA databases traditionally rely on static locus-count thresholds to determine search eligibility and report matches. While computationally straightforward, these rigid criteria routinely discard high-value investigative leads from degraded forensic profiles while simultaneously permitting adventitious matches when common alleles are involved. To overcome the limitations of static rules, this study introduces an automated framework for dynamic likelihood ratio (LR) thresholding. **Methods:** Utilizing a Fast Fourier Transform (FFT) algorithm, the system calculates the Probability Mass Function (PMF) for any specific combination of shared loci in real-time, natively incorporating the Balding–Nichols model to account for population substructure. Instead of applying an arbitrary locus count or fixed LR cutoff, the framework defines admissibility based on a user-defined maximum upper bound of acceptable false positives at a specified confidence (probability) level (e.g., 95%). **Results:** This empowers database custodians to precisely predict and adapt their search criteria to match an acceptable administrative workload, dynamically adjusting the required LR threshold to the exact size of the searched database. This approach was validated through massive-scale empirical simulations across five reference population groups. Receiver Operating Characteristic (ROC) and Poisson distribution analyses reveal that static thresholds inevitably collapse under the multiplicity effect of large-scale comparisons; for instance, a static locus rule that maintains safety within a small DNA database yields an unmanageable false positive risk when scaled to larger DNA databases or international networks like the Prüm DNA Exchange. **Conclusions:** By explicitly coupling the decision threshold to the database size and the genetic rarity of the evidence, this dynamic framework provides a mathematically rigorous and scalable solution. Most notably, it identifies rare, low-locus matches that static rules typically discard, offering a method to maintain a predefined expected false positive rate.

## 1. Introduction

DNA profiling is an invaluable tool in forensic investigations, assisting in the identification and exclusion of individuals from crime scenes. The analysis of Short Tandem Repeats (STRs) has been widely employed in DNA database searches to establish hits between potential suspects and/or convicted individuals, crime scene samples, missing persons and unidentified human remains, depending on applicable national legislation [1]. Most operational DNA databases rely on locus-count–based eligibility rules at multiple stages of the process, including profile upload (number of typed loci), database searching (number of shared loci), match reporting (number of corresponding loci), and storage criteria for future comparison. These criteria are widely adopted, notably by the approximately 70 countries reporting the existence of a national DNA database [2].

Historically, major DNA databases relied exclusively on strict minimum locus-count thresholds for profile uploads and match reporting. For example, the United States originally required a minimum of 10 of the 13 CODIS Core Loci to initiate an automated search on the National DNA Index System (NDIS) [3,4]. Similarly, at the international level, both the European Union’s Prüm framework [5] and the INTERPOL DNA Database mandate a minimum of six fully matching loci (excluding amelogenin) before a hit response is provided between member countries.

Recognizing the limitations of relying solely on locus counts, several jurisdictions have since transitioned to hybrid criteria that incorporate probabilistic measures. In 2016, the United States revised its NDIS procedures to accept profiles with a minimum of only eight loci, provided they demonstrate a random match probability (RMP) of at least one in ten million [3]. This shift aligns with recommendations from the ENFSI DNA Working Group, which highlighted RMP as a more appropriate metric for database inclusion than locus counts alone [6]. Comparable hybrid approaches, combining reduced locus thresholds with RMP requirements, are now utilized to report investigative leads in several countries across the Americas, including Brazil, Mexico, and Colombia [7,8].

While strict locus thresholds ensure highly specific automated workflows, they inadvertently exclude matches with significant intelligence value, especially in casework involving limited or degraded DNA. Simulation studies using a Swiss DNA database have demonstrated that relaxing locus thresholds can substantially increase the detection of genuine matches, albeit with a concomitant increase in false positives if not appropriately controlled [9]. To address this limitation, some jurisdictions may permit exceptional “keyboard searches,” defined as manual, one-time database queries performed outside routine automated searches. These searches are typically reserved for urgent or challenging cases that fail to meet standard upload requirements. Although keyboard searches provide an essential investigative safeguard, they are highly resource-intensive, requiring manual profile assessment, administrative authorization, and expert interpretation of candidate matches. As database volumes and case backlogs continue to grow, this reliance on manual intervention creates a significant operational bottleneck, effectively restricting such searches to only the most serious or high-profile cases.

In situations where other investigative avenues have been exhausted, a DNA match involving a limited number of shared loci but supported by a strong LR may nevertheless provide critical intelligence, either by directing attention to a person of interest or corroborating existing hypotheses [10]. To overcome the limitations of binary locus-count rules, the forensic community has increasingly adopted probabilistic frameworks, most notably the likelihood ratio, to quantify the strength of database matches. Software tools that either possibly use probabilistic genotyping (e.g., DBLR, ProbRank) or operate without it (e.g., SmartRank) enable database coincidences to be ranked by LR rather than by locus count alone, facilitating the interpretation of matches involving mixtures or degraded profiles where traditional criteria fail [11,12,13,14,15]. By explicitly incorporating population genetic models, these approaches capture the rarity of observed genotypes and reveal an important empirical reality: pairs of DNA profiles that share as few as three to five matching loci may yield higher LRs than pairs sharing six or more matching loci when rare alleles are involved [6]. However, a substantial operational gap remains. As with keyboard searches, most LR-based tools operate as post-search evaluators rather than as fully automated decision engines, requiring manual data preparation and interpretation. This limits their integration into high-throughput national database workflows. Moreover, while these tools produce numerical LR values, they do not define objective decision thresholds for distinguishing meaningful investigative leads from adventitious matches. As a result, practitioners must still decide whether an LR of, for example, 10,000 is sufficient for reporting, perpetuating inconsistency and reinforcing dependence on expert judgment and manual review.

The challenge of defining LR thresholds for reporting database matches has been widely discussed, yet no universal standard has emerged. Early work by Gill et al. highlighted that LR cutoff selection inherently balances the risk of false exclusions against the risk of adventitious matches, advocating laboratory-specific thresholds supported by empirical validation of sensitivity and specificity [16]. Bright et al. demonstrated the feasibility of direct database searching using an LR framework, showing that a threshold of approximately 1 million could effectively filter out adventitious hits even in complex mixtures [17]. More recent studies using SmartRank and ProbRank have explored pre-defined LR thresholds to control candidate volumes in large national databases [11,13,14]. These approaches, however, rely on static thresholds applied irrespective of the database size or the specific locus composition of the stain. Consequently, they fail to mathematically account for the problem linked to multiple comparisons, the statistical phenomenon where the sheer volume of pairwise comparisons in expanding databases inevitably surfaces coincidental matches [18]. A static threshold, whether a six-locus minimum or a fixed LR of 10,000, that successfully suppresses adventitious matches in a local index of 100,000 profiles will inevitably collapse under the statistical weight of very large DNA databases or of an international network like Prüm, generating an unmanageable operational burden of false positive hits for DNA database custodians. When laboratories do not report matches that fall below locus-count thresholds, even if those matches generate strong LR values, investigations may be delayed, and potentially solvable cases may remain unresolved. To fully overcome this limitation, probabilistic decision-making must be integrated into routine, automated DNA database searches, enabling the identification of rare but meaningful matches while simultaneously neutralizing the multiplicity effect as databases expand to ease the administrative workload for database managers and practitioners.

**Figure 1:**
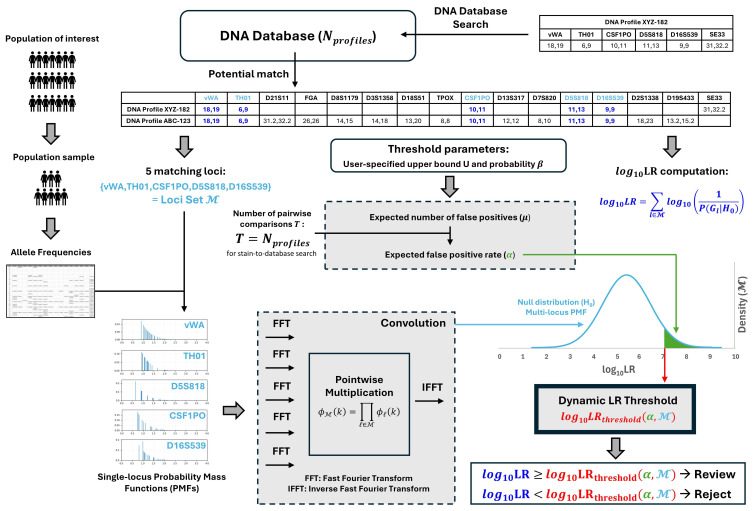
A Fast-Fourier-Transform-Based Dynamic Likelihood Ratio Framework. Allele frequencies are derived from a representative population sample to generate single-locus Probability Mass Functions (PMFs) for the loci of interest. A query profile is compared against a DNA database containing N_profiles_ profiles to identify potential matches. In this example, a match is identified based on 5 shared loci. To evaluate the significance of the match, a case-specific null distribution H_0_, representing the likelihood ratios of unrelated individuals, is generated for the specific set of matching loci. This is achieved by the convolution of the corresponding single-locus PMFs using Fast Fourier Transform (FFT) and Inverse Fast Fourier Transform (IFFT) techniques. The observed ${log}_{10}LR$ is compared against the ${{log}_{10}LR}_{threshold}$. As shown in the workflow, this threshold is derived by setting a probabilistic upper bound ($U_{\beta }$, calculated here via the 95% upper confidence bound of the Poisson distribution) on the expected number of false positives accepted by the DNA operator. This threshold is unique for the set of loci M and the number of pairwise comparisons T performed in the search and will determine whether the DNA match should be reviewed or rejected automatically, depending on the log_10_LR calculated from the allele values of the pair of DNA profiles.

To address these challenges, this study introduces and validates a proof-of-concept framework for automated, dynamic LR decision thresholds in DNA database searching (Figure 1). Unlike conventional static rules, our method derives match-specific thresholds that adapt to both the genetic rarity of the matched loci and the exact size of the searched database. By employing a Fast Fourier Transform strategy to compute probability distributions under the Balding–Nichols model, the framework allows database custodians to set a precise, manageable limit by specifying an upper bound (U_β_) on the number of acceptable false positives at a chosen confidence level β. We empirically validate this approach through massive-scale simulations across five global population groups, utilizing Receiver Operating Characteristic (ROC) and Poisson distribution analyses. Ultimately, we demonstrate that while static thresholds inevitably collapse under the statistical weight of expanding databases, this dynamic framework successfully uncouples the False Positive Rate (FPR) from database size, maximizing the extraction of investigative intelligence while strictly minimizing the administrative burden of adventitious leads.

## 2. Materials and Methods

### 2.1. Reference Populations and Allele Frequencies

Five reference populations were used in this study. Four are continental reference populations (African American, Asian, Caucasian, and Hispanic) published by the National Institute of Standards and Technology (NIST), comprising a total of 1036 individuals [19]. The fifth reference population is a global dataset concatenated and curated by INTERPOL, drawn from 182,999 individuals representing 369 distinct populations, abbreviated as INTERPOL-WAF (for Worldwide Allele Frequencies) [20]. The raw data for this global dataset, representing 2,407,482 typed alleles, underwent a multi-stage curation process to resolve nomenclature inconsistencies and frequency anomalies, resulting in the most extensive curated reference population dataset to date [20]. Allele frequencies for 24 autosomal STR loci (CSF1PO, D10S1248, D12S391, D13S317, D16S539, D18S51, D19S433, D1S1656, D21S11, D22S1045, D2S1338, D2S441, D3S1358, D5S818, D6S1043, D7S820, D8S1179, FGA, PENTAD, PENTAE, SE33, TH01, TPOX, and vWA) were gathered for the five reference populations.

### 2.2. Simulated DNA Profiles and Database Generation

To evaluate the performance of the various thresholding strategies, in silico DNA databases and degraded stains were generated using empirical allele frequencies from the five reference populations. To account for sampling bias and the potential presence of rare variants not observed in the reference set, a minimum allele frequency $p_{\text{\_}min}$ was calculated for each locus. This was derived using the conservative formula 5/2N, where N is the number of individuals typed for the specific locus. Supplementary Table S1 recapitulates the $p_{\text{\_}min}$ values for each locus and each reference population. A critical requirement for this simulation was to ensure that the generated populations correctly reflected the subpopulation structure assumed by the LR calculations (the Balding–Nichols model, with θ=0.01) [21]. Generating profiles directly from allele frequencies assuming standard Hardy–Weinberg Equilibrium (HWE) artificially maximizes population diversity, thereby underrepresenting the true rate of coincidental matches. To resolve this, population structure was modeled using a Dirichlet distribution [22]. The Dirichlet distribution is a multivariate probability distribution used to model variables that must sum to one, such as allele frequencies at a given locus. By using the general population’s allele frequencies as baseline parameters, the distribution generates new, skewed subpopulation frequencies that simulate random genetic fluctuation. This mathematically limits genetic diversity and introduces Identity by Descent (IBD) alleles, accurately simulating the genetic drift of an isolated subpopulation. To simulate degraded crime stains, full profiles were randomly selected from the generated database. Degradation was simulated by randomly dropping loci until a specific target length (ranging from 3 to 9 loci) was reached.

Traditionally, artificial degradation is carried out to mimic the biological process leading to a preferential drop of loci with longer amplicons in DNA typing kits over shorter amplicons. The random dropping of loci was chosen over the more biologically correct degradation process to include the typical sets that are regularly observed (mirroring the true biological process) but also sets of loci that originate from exotic or old typing kits, as well as composite DNA profiles, which may be obtained by combining the results of two independent typing kits.

A synoptic table compiling all simulations performed in this study can be found in Supplementary Table S2.

### 2.3. Mathematical Framework for Match Probabilities and Genotype Frequencies

We assume that the match is performed on a set of common loci $M=\left\{l_{1},l_{2},\ldots ,l_{m}\right\}.$ For each locus l, we define the set $G^{l}$ of all genotypes $g_{ij}=\left(a_{i},a_{j}\right)$ with alleles $a_{i},a_{j}\in \;L^{l}$ and i≤j, where $L^{l}$ is the ladder, i.e., the set of alleles for locus l for the current population. With allele frequencies $p_{i}$ for allele $a_{i}$ for locus l and taking population sub-structuring and the effect of shared ancestry θ into account according to the Balding–Nichols model [21], we have the following single-locus results:

- Genotype Frequencies

The probability that a random, unrelated person has a certain genotype g is

• For homozygotes $g_{ii}=(a_{i},a_{i})$:
  (1) $$P\left(g_{ii}\right)=\theta p_{i}+\left(1-\theta \right)p_{i}^{2}$$
• For heterozygotes $g_{ij}=\left(a_{i},a_{j}\right)$:
  (2) $$P\left(g_{ij}\right)=2\left(1-\theta \right)p_{i}p_{j}$$

B. Genotype Frequencies of Unrelated Persons (*H*_0_)

The defense hypothesis (*H*_0_)—“Given that we have seen a genotype g at the crime scene, the probability that a random, unrelated person has this same genotype g” is

• For homozygotes:
  (3) $$P\left(g_{ii}|g_{ii},H_{0}\right)=\frac{\left(3\theta +\left(1-\theta \right)p_{i}\right)\left(2\theta +\left(1-\theta \right)p_{i}\right)}{\left(1+\theta \right)\left(1+2\theta \right)}$$
• For heterozygotes:

(4) $$P\left(g_{ij}|g_{ij},H_{0}\right)=\frac{2\left(\theta +\left(1-\theta \right)p_{i}\right)\left(\theta +\left(1-\theta \right)p_{j}\right)}{\left(1+\theta \right)\left(1+2\theta \right)}$$

which is NRC II Recommendation 4.10 [21].

C. Probability of a Match (*H*_1_)

The prosecutor’s hypothesis (*H*_1_)—“Given that we have seen a genotype h at the crime scene, the probability that the suspect who is the actual donor of the source has genotype g” is

(5) $$P\left(g|h,H_{1}\right)=\left\{\begin{array}{c} 1⟺g=h\\ 0⟺g\neq h \end{array}\right.$$

D. Likelihood Ratio

The likelihood ratio LR is the ratio between the probabilities of finding the genotype under the two hypotheses. For equal genotypes g, the likelihood ratio is

(6) $$LR\left(g\right)=\frac{P\left(g|g,H_{1}\right)}{P\left(g|g,H_{0}\right)}=\frac{1}{P\left(g|g,H_{0}\right)}$$

Since LR(g,g) is a function of a single variable, we define for convenience the equal-genotype likelihood ratio LRE as

(7) $$LRE\left(g\right)=LR\left(g,g\right)$$

For homozygotes, this results in

(8) $$LRE\left(g_{ii}\right)=\frac{\left(1+\theta \right)\left(1+2\theta \right)}{\left(3\theta +\left(1-\theta \right)p_{i}\right)\left(2\theta +\left(1-\theta \right)p_{i}\right)}$$

For heterozygotes, this is

(9) $$LRE(g_{ij})=\frac{\left(1+\theta \right)\left(1+2\theta \right)}{2\left(\theta +\left(1-\theta \right)p_{i}\right)\left(\theta +\left(1-\theta \right)p_{j}\right)}$$

### 2.4. Total Log-Likelihood Ratio

To estimate the FPR in a match with a certain ${LR}_{threshold}$, we are interested in the probability that a match between two unrelated individuals in the population would achieve a total LR greater than or equal to the ${LR}_{thresholds}$ for that specific match or, equivalently, that the total logLR is greater than or equal to the $log{LR}_{thresholds}$.

The total log-likelihood ratio ${logLR}_{M}$ for a profile $pr=\left\{g^{1},g^{2},\ldots ,g^{m}\right\}$ consisting of a set M of independent loci can be obtained by adding the per-locus log-likelihood ratios for each locus l,

(10) $${logLR}_{M}(pr)=\sum_{l\in M}{log}_{10}({LRE}_{l}(g^{l}))$$

where ${LRE}_{l}$ is the equal-genotype likelihood ratio for locus l.

If we view the genotypes of random persons as a random variable, then the log-likelihood ratios per locus and the total log-likelihood of unrelated persons are random variables as well. The distribution of the total-log-likelihood ratio can be obtained as a highly precise discretized approximation from the convolution of the distributions of the log-likelihood ratios per locus. The distributions of the log-likelihood ratios per locus can be obtained from the probabilities of matching genotypes of random persons.

### 2.5. Single-Locus Probability Mass Function (PMF) and Total Log-Likelihood Ratio PMF

The probability that two unrelated random persons have an equal genotype *g* at a given locus l is

(11) $$P_{l}^{\mathrm{equal}}\left(g\right)=P\left(g|g,H_{0}\right)P\left(g\right)$$

Note that since we are only considering equal genotypes, the total probability is

(12) $$P_{tot,l}^{equal}=\sum_{g\in G^{l}}P_{l}^{equal}\left(g\right)\leq 1$$

With a matching genotype *g* at a given locus, the log likelihood ratio at this locus is

(13) $${logLR}_{l}\left(g\right)=\log_{10}\left({LRE}_{l}\left(g\right)\right)$$

The PMF of a single locus log-likelihood ratio, restricted to nonnegative logLR values x ≥0, is

(14) $$P_{logLR_{l}}\left(x\right)=\sum_{g\in G^{l}:logLR_{l}\left(g\right)=x}P_{l}^{equal}(g)\;\;$$

It should be noted that because of the restriction on equal genotypes $\sum_{x}P_{logLR_{l}}\left(x\right)<1$, we are only looking at a part of the distribution.

The PMF of the total log-likelihood ratio, restricted to nonnegative logLR values, is formally obtained from the single-loci log-likelihood PMFs. For instance, if the matching loci set M consists of locus 1,…, M, then the probability of having $logLR_{M}=x$ is obtained by the convolution

(15) $$P_{logLR_{M}}\left(x\right)=\sum_{x_{1},\ldots ,x_{M}:x_{1}+\ldots +x_{M}=x}P_{logLR_{1}}\left(x_{1}\right)\ldots P_{logLR_{M}}\left(x_{M}\right)$$

### 2.6. Fast Fourier Transform (FFT)-Based Convolution for Multi-Locus Log-Likelihood Ratio PMF

Convolutions of independent single-locus log-likelihood ratio distributions can be computed efficiently using the FFT [23,24]. The computational advantage of using convolution in the context of log-likelihood distributions has been noted earlier by Perlin [25]. To apply the FFT, the single-locus log-likelihood ratio PMFs are first mapped onto a common grid by defining

(16) $$n=round\left(\;\frac{x}{\Delta }\;\right)$$

where Δ denotes the chosen bin width. For each locus, the discretized single-locus log-likelihood ratio PMF is

(17) $$P_{{logLR}_{l}}^{\Delta }\left(n\right)=\sum_{x:round\left(x/\Delta \right)=n}P_{logLR_{l}}\left(x\right)$$

This PMF is transformed into characteristic function $\phi_{l}$ in the frequency domain using the FFT:

(18) $$\phi_{l}\left(k\right)=\sum_{n=0}^{N-1}P_{{logLR}_{l}}^{\Delta }\left(n\right)\cdot e^{-i2\pi \frac{k}{N}n}$$

where $N=2^{16}$ represents the total array length, k = 0, …, N−1 and i is the imaginary unit.

The characteristic function of the ${LR}_{total}$ distribution $\phi_{total}$ for a specific match configuration is obtained by the point-wise multiplication of the transformed PMFs of the matching loci l∈M:

(19) $$\phi_{M}\left(k\right)=\prod_{l\in M}\phi_{l}\left(k\right)$$

The final discretized total log-likelihood ratio PMF $P_{{logLR}_{tot}}^{\Delta }$ is obtained via the Inverse Fast Fourier Transform (IFFT):

(20) $$P_{{logLR}_{M}}^{\Delta }\left(n\right)=\frac{1}{N}\sum_{k=0}^{N-1}\phi_{M}(k)\cdot e^{i2\pi \frac{k}{N}n}$$

In this paper, we chose the bin width ∆ = 0.001. This means that a ${log}_{10}(LR)$ of 1.000 and 1.0004 are treated as the same value, while 1.001 is the next adjacent bin. To prevent circular convolution artifacts (aliasing), the arrays are zero-padded to provide sufficient space for the signal to expand during convolution. For a 24-locus system where the total log-likelihood ratio $logLR_{tot}$ can reach 40.0, a range of at least 40,000 bins is required (40/0.001). Since the FFT is most computationally efficient when the array size is a power of two, we utilize an array length of 2^16^ = 65,536 [26].

### 2.7. Calculation of the Expected False-Positive Rate α from the logLR Threshold and Vice Versa

Now that we can efficiently compute the distribution of the total log-likelihood ratios under $H_{0}$ for any loci set M, we can, given a threshold ${LR}_{threshold}$, directly compute the expected FPR α. This value represents the probability that an unrelated individual in the population would achieve a total LR equal to or greater than the LR threshold.

(21) $$\alpha ({LR}_{threshold},M)=P\left(LR_{M}\geq LR_{threshold}\right)\approx \sum_{k=round\left(logLR_{threshold}/\Delta \right)}^{N-1}P_{{logLR}_{M}}^{\Delta }\left(k\right)$$

in which the approximation is only due to the discretization.

The formula shows that for a given locus set M, an LR threshold implies an expected FPR $\alpha ({LR}_{threshold},M)$. But the converse also holds: a given locus set M and expected FPR α implies an LR threshold ${LR}_{threshold}$(α,M).

This provides two approaches to setting an LR threshold. The conventional way is to fix the LR threshold and apply it regardless of the set of overlapping loci of the compared profiles. This approach results in an expected FP rate, which will depend on the loci set. The approach that we propose in this paper is to perform the matching with a fixed expected FP rate α and compute LR thresholds depending on the overlapping loci sets. This results in an expected FP rate that is independent of the loci set.

### 2.8. Dynamic Thresholds Based on Acceptable Risk Upper Bounds (U_β_) in Database Search

So far, the analysis has considered a single comparison. In a conventional database search, multiple comparisons are performed during a search. Let T represent the total number of pairwise comparisons executed during a search and $N_{Profiles}$ the number of profiles in the database (i.e., the size of the DNA database):

For a database-to-database search (checking if any profile in a database of $N_{Profiles}$ matches any other profile in the same database):

(22) $$T=\frac{N_{Profiles}(N_{Profiles}-1)}{2}$$

For a stain-to-database search (one crime stain searched against a database of $N_{Profiles}$):

(23) $$T=N_{Profiles}$$

In a search with T > 1 comparisons, the number of false positives is a random variable X. If in each pairwise comparison the FPR is fixed to be α, the expected number of false positives, μ=E[X], follows directly

(24) μ=α·T

The probability β of observing at most U false positives is given by the cumulative probability

(25) $$\beta =P\left(X\leq U\right).\;$$

In practice, α is very small so that the number of false positives X is well approximated by a Poisson (μ) distribution. The cumulative probability is given by the cumulative distribution function (CDF)

(26) $$P\left(X\leq U\right)=F\left(U;\mu \right)$$

where

(27) $$F\left(U;\mu \right)=\sum_{k=0}^{U}\frac{e^{-\mu }\mu^{k}}{k!}$$

To summarize, from a fixed FPR α and the number of comparisons T, we can infer the expected number of false positives μ. Then, given μ, we can specify any number U and compute the probability β that the observed number of false positives does not exceed U.

### 2.9. Dynamic Thresholds Based on Acceptable Risk of Number of False Positives in Database Search

Our proposed framework operates in reverse. The laboratory defines an upper bound U on the number of false positives, together with a required probability level β, representing the probability that the number of false positives does not exceed U. Then, by inverting the relation β= F(U;μ), an acceptable expectation of false positives $\mu =F^{-1}\left(U,\beta \right)$ is computed. The required per-comparison false positive probability α follows from the number of comparisons performed in the search:

(28) $$\alpha =\frac{\mu }{T}$$

Once α is established, the framework then needs to determine for each pairwise comparison the overlapping loci set M so that the dynamic threshold ${LR}_{threshold}$(α,M) can be computed and applied. Note that ${LR}_{threshold}$(α,M) only needs to be computed once per overlapping loci set M, since the result can be reused for all pairwise comparisons with the same overlapping loci.

### 2.10. Performance Assessment

To evaluate the discriminative power and operational viability of the proposed framework, performance was assessed using a two-tiered approach: ROC analysis and Poisson risk modeling. Although users may specify admissibility through an upper bound U on the number of false positives at probability level β, they may also just specify the expected number of false positives μ. We took this approach in some of the simulations and performance assessments that are presented in the next sections. This was done for convenience because with μ, only a single parameter needs to be specified, and μ is the natural parameter of the Poisson distribution.

However, since this reasoning relies on the Poisson assumption for the number of false positives, the adequacy of this assumption was evaluated in one of the simulation experiments. In particular, the simulated numbers of false positives were compared not only with the expected value implied by μ but also with the corresponding 95% lower and upper bounds predicted by the Poisson distribution.

To illustrate this relationship, Table 1 demonstrates how a laboratory’s desired upper bound (U_β_) translates into the operational expectation parameter μ used by the framework.

For example, for μ=10, in 95% of the searches, the number of false positives will be 15 or lower, and in 99% of the searches, the number of false positives will be 18 or lower. On average, across all the searches, the average of false positives will be 10.

#### 2.10.1. Receiver Operating Characteristic Analysis

We assessed the performance of our predictions with ROC curves and their corresponding Areas Under the Curve (AUCs) across three distinct match-filtering methods (locus-count threshold, static LR threshold and dynamic LR threshold). To generate the discrete true positive (TP) and false positive (FP) datasets required for the ROC curves, large-scale computational simulations were performed. A synthetic reference database of 100,000 profiles was generated, alongside sets of 1000 degraded crime scene stains for each locus count, ranging from highly degraded (3 loci) to moderately degraded (7 loci). These stains were cross-searched against the database to calculate the true positive rate (TPR) and FPR at every threshold step for each of the three methods. To ensure the robustness and universal applicability of the findings, this entire simulation was executed independently across five distinct population frequency datasets. The final coordinates for the ROC curves and their corresponding AUCs were obtained across all five populations.

#### 2.10.2. Operational Risk Modeling

Because ROC analysis evaluates discriminative power independently of database size, it fails to capture the multiple comparisons problem inherent in expanding databases. Therefore, a secondary performance assessment was conducted to evaluate the operational burden of each method. Utilizing the baseline coincidental match rates observed during the simulations, the expected number of false positives μ was scaled to simulate real-world database sizes ranging from 100,000 to 50 million profiles. The Poisson probability formula

(29) $$P\left(X\geq 1\right)=1-e^{-\mu }$$

was then applied to calculate the exact probability of encountering at least one adventitious match during a single casework search. This allowed for a direct quantitative comparison of how each filtering method withstands the statistical weight of database expansion. However, this linear extrapolation to 50 million profiles has limitations. It assumes all database entries are independent, ignoring complex population substructures and the presence of relatives, which exponentially increases the risk of adventitious matches via Identity by Descent. Additionally, at only 3 or 4 loci, a database of this size begins to saturate the available genotype space. Therefore, these extrapolated probabilities should be viewed as theoretical baselines for comparing filtering methods, rather than exact operational predictions.

#### 2.10.3. Evaluation of Continuous Approximation Models

To empirically assess the structural limitations of continuous risk estimation models, the discrete PMFs generated by the FFT framework were compared against continuous Gaussian approximation. For any given multi-locus combination, the mathematical mean and variance were calculated from the underlying single-locus PMFs. These parameters were used to generate a continuous Normal function. A spatial gap analysis was then computationally performed across combinations ranging from 2 to 10 loci to map the exact locations and frequencies of significant mathematical discontinuities (defined as intervals of zero probability wider than $0.04\;{log}_{10}LR$) across the generated distributions.

### 2.11. Simulation of Familial DNA Profiles

To evaluate the impact of familial relationships on the Dynamic LR framework, we employed a rigorous Monte Carlo simulation based on the fundamental genetic principles of Mendelian inheritance and IBD. The simulation was designed to mathematically recreate the exact genetic sharing expected between a crime stain donor and their close relatives. Once the baseline donor profile was established, the corresponding relative profiles were simulated locus-by-locus using the following IBD probabilities. For parent–child relationships, the algorithm forced the relative to share exactly one allele IBD with the donor at every locus. For full sibling relationships, at any given locus, the simulation applied standard Mendelian probabilities to determine inheritance: a 25% probability of sharing both alleles (IBD = 2), a 50% probability of sharing one allele (IBD = 1), and a 25% probability of sharing zero alleles by descent (IBD = 0). Alleles not shared by descent were drawn independently from the frequency pool. Once the reference database, the stains, and the corresponding relative profiles were generated, we simulated degraded casework by truncating the profiles to partial lengths of 3, 4, 5, and 6 loci. We then performed iterations of pairwise cross-comparison of all 10,000 partial stains against the 100,000-profile database, amounting to one billion physical profile comparisons.

### 2.12. Computational Pipeline

The computational pipeline for these comparisons was implemented in Python (version 3.13.12). We utilized the pandas and NumPy libraries for efficient data manipulation and large-scale array operations, alongside SciPy (specifically scipy.fft) for Fast Fourier Transform calculations. All computations were executed on a workstation running Windows 11, equipped with an 11th Gen Intel Core i5-1145G7 processor and 8 GB of RAM (Intel Corporation, Santa Clara, CA, USA).

## 3. Results

### 3.1. Characteristics of Allele Frequencies from the 5 Population Datasets

Prior to simulation, the population datasets were analyzed to determine genetic diversity and frequency distributions across the 24 autosomal STR loci. As shown in Supplementary Table S1, the data exhibits a high level of polymorphism across all five reference populations. The number of rare alleles—defined as those with a frequency lower than the minimum threshold p_min obtained using the conservative 5/2N rule, where N is the number of individuals typed for a specific locus—is reported for each population. All four continental populations exhibited a comparable number of rare alleles across the 24 STR loci (101 for NIST-African American, 69 for NIST-Asian, 79 for NIST-Caucasian, and 94 for NIST-Hispanic). In contrast, the INTERPOL-WAF dataset contained 379 rare alleles, which is expected given the significantly larger number of individuals N per locus. Properly defining these parameters (p_min and N) is essential to ensure that the subsequent RMP simulations align with established forensic standards. Specifically, the implementation of the 5/2N rule reflects the minimum allele frequency threshold recommended by the National Research Council [21] and the Scientific Working Group on DNA Analysis Methods (SWGDAMs) to account for sampling variation [27]. By adhering to these guidelines, our simulations accurately mirror the statistical constraints applied in routine forensic casework and database searches.

### 3.2. Quantitative Assessment of RMP Distributions

To investigate the relationship between the number of overlapping loci and the resulting discriminating power, RMPs were simulated using the allele frequencies from the five populations, applying a θ=0.01. For each condition (3 to 8 loci), 100,000 profiles were generated by randomly sampling loci and alleles, with p_min corrections applied for any frequency below the threshold.

**Figure 2:**
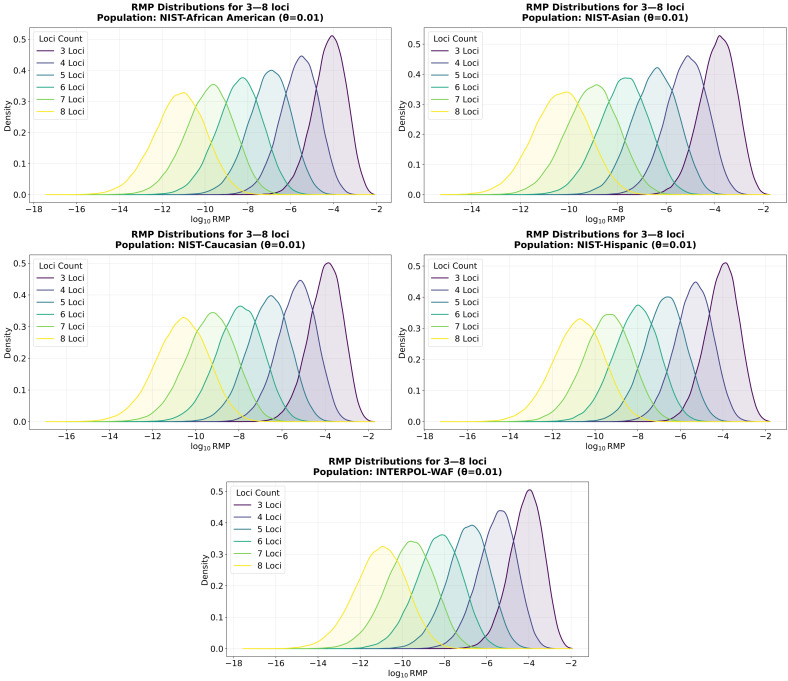
Multi-locus Random Match Probability (RMP) distributions for varying matching loci counts (3–8). Distributions were generated using 100,000 simulated profiles per condition (3–8 loci), sampled from global allele frequencies. RMPs were obtained for the five reference populations. As the number of loci increases, the distributions shift toward more discriminating values log_10_(RMP), reflecting the increased evidentiary weight. A significant overlap exists between all conditions, confirming that discriminatory power is an inherent property of the specific alleles present rather than a simple function of locus count.

The data in Figure 2 and Supplementary Table S3 confirms a substantial mathematical overlap between the RMP distributions of profiles with different loci counts. For example, the distribution overlap between five-locus and six-locus matches is approximately 50% (ranging from 49.39% to 52.75% across the evaluated populations). This indicates that a highly informative five-locus match will frequently provide a higher evidential value than a less informative six-locus match. Furthermore, this overlap is not restricted to adjacent groups; the distribution overlap between four-locus and six-locus matches is approximately 17% (ranging from 15.19% to 18.29% across the evaluated populations) while the distributions for six-locus and eight-locus matches still share between 21.91% and 25.29% of their area. A similar phenomenon of stochastic overlap was noted in a previous publication simulating the Swiss national DNA database [9]. The fact that these overlap percentages remain remarkably consistent across five independent reference populations confirms that this overlap is an inherent mathematical property of STR probabilities rather than an artifact of a specific population structure.

### 3.3. Convergence of FFT and Monte Carlo Methods

Monte Carlo simulations are often the method of choice for random sampling to generate synthetic data based on probability distributions and have been applied in forensic genetics [28,29,30,31]. Monte Carlo simulations are stochastic: they estimate the distribution by randomly generating synthetic profiles. While effective for general trends, including for application in forensic genetics, they suffer from two major limitations in this context. Firstly, billions of iterations are required to accurately model the extreme tails of the distribution where the rarest and most highly discriminating genotypes reside. Standard Monte Carlo simulations often fail to sample these rare events sufficiently to form a smooth curve, leading to gaps in the risk assessment for high-value evidence. Secondly, our proposed strategy requires a tailored risk assessment for the specific set of loci (e.g., a specific combination of loci shared between the two profiles). With millions of possible locus combinations, running a bespoke Monte Carlo simulation for every incoming search query is computationally prohibitive. In contrast, the FFT approach is deterministic as it treats the genotype probabilities of each locus as a PMF and uses mathematical convolution to calculate the distribution of likelihood ratios across the entire combinatorial space. This allows for the precise determination of the risk, including within the extreme tails, without the need for real-time simulation. The method effectively captures the probabilistic landscape without the computational bottleneck of live simulation, making it the appropriate computational strategy for a dynamic threshold system.

**Figure 3:**
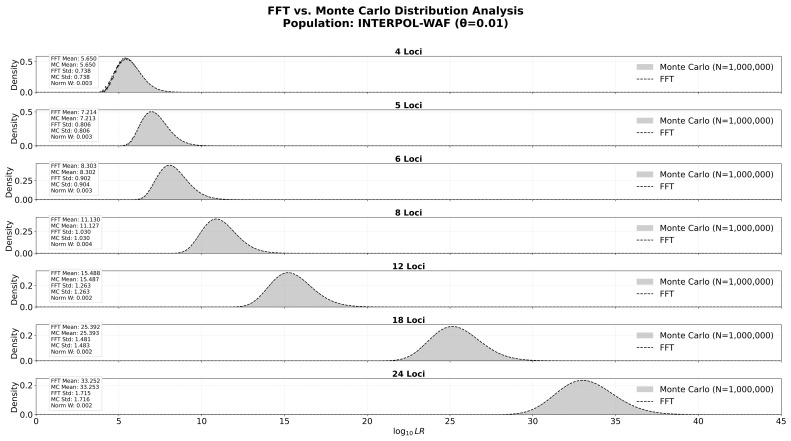
Comparison of theoretical probability density functions generated via FFT (dashed lines) and empirical distributions obtained from 1,000,000 Monte Carlo trials (gray histograms). The simulations have been carried out on sets of 4-, 5-, 6-, 8-, 12-, 18- and 24-locus from the INTERPOL-WAF population.

To demonstrate the interchangeability of the two methods, we analyzed the convergence of the distributions generated by the FFT method against 1,000,000 empirical Monte Carlo trials for varying profile complexities (4 to 24 loci), based on the INTERPOL-WAF population. As demonstrated in Figure 3, the convergence between the two models is exceptionally high across all tested parameters.

The mean ${log}_{10}LR$ values for the FFT and Monte Carlo methods remain virtually identical at every level of complexity. Similarly, the standard deviation (STD) values show a consistent match, confirming that the FFT method correctly captures the genetic variance and spread of the population’s match strength. To quantify the similarity between the two probability distributions, we utilized the Normalized Wasserstein Distance (Norm W-Dist) [32,33]. This metric represents the effort required to transform the empirical histogram into the theoretical curve, normalized by the distribution’s spread. Across all tests, the Norm W-Dist remained consistently low (ranging from 0.002 to 0.004). In statistical modeling, a normalized deviation of this magnitude is considered negligible and indicates that the minor differences observed are attributable to the stochastic sampling noise of the Monte Carlo method rather than any systemic error in the FFT algorithm. These results validate the use of the FFT library as a robust, mathematically precise tool for determining search thresholds in forensic databases.

### 3.4. Locus-Specific Informational Content and Convolution

**Figure 4:**
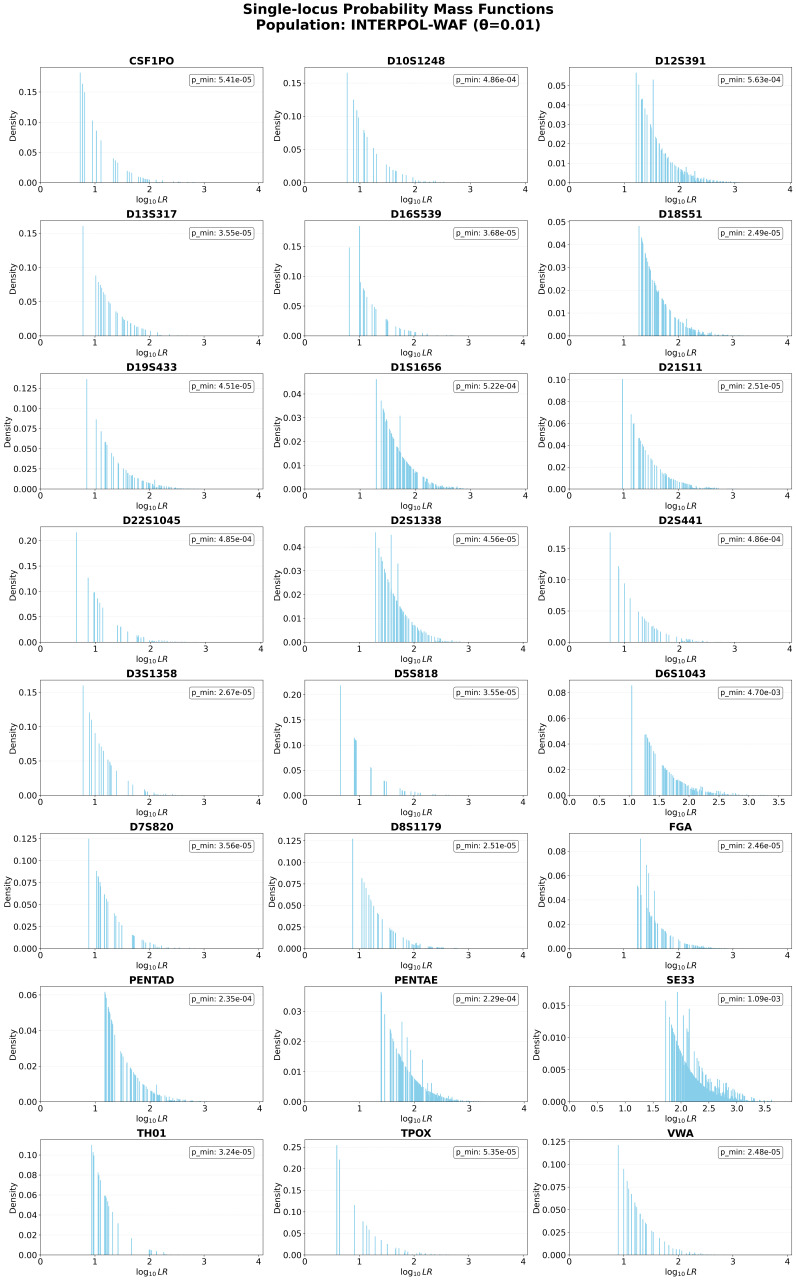
Single-locus Probability Mass Function (PMF) for 24 loci. Each subplot represents the PMF of log_10_LR for a single locus, calculated from the INTERPOL-WAF with θ=0.01. The discrete bars represent the specific evidential weights of all possible genotype combinations.

The construction of the FFT library allowed for a high-resolution analysis of the informational heterogeneity across the standard 24-STR multiplex. While most search models treat forensic loci as a homogenous set, the PMFs generated in this study reveal significant disparities in the discriminatory power and stochastic background of individual genetic markers. As illustrated in Figure 4, the distribution of match strengths is inherently discrete. Each spike in the distribution represents a specific genotype combination, with the height of the spike corresponding to the genotype frequency in the population.

A critical observation is the presence of significant probabilistic gaps, log_10_LR values that are physically impossible for a given locus (typically observed for TPOX and CSF1PO). Conversely, some loci (e.g., D12S391, SE33, or PENTAE) display clumping, where two or three distinct genotypes fall into the same evidential bin. Summing these probabilities leads to pronounced spikes in the PMF, highlighting how the discrete nature of alleles affects the observed distribution. At any single locus, there are only a limited number of possible allele combinations (for example, a locus with 10 alleles produces exactly 55 possible genotypes). As loci are convolved together (as shown in Supplementary Figure S1), the number of possible combinations grows exponentially. This combinatorial expansion is why multi-locus distributions begin to approximate smooth curves, as the gaps are progressively filled by the sheer variety of genotypes from other loci. Traditional continuous models (such as Gaussian distributions) rely on this expansion, assuming that this smoothing inevitably leads to a perfectly symmetrical, infinite bell curve [34].

To empirically confirm the structural limitations of this symmetry assumption, we computationally evaluated the statistical shape and operational tail risk of 10,000 randomly generated six-locus profiles, comparing the discrete FFT distribution against a mathematically optimized continuous Gaussian baseline (Supplementary Table S4). Across all 10,000 simulated six-locus profiles, the discrete PMF exhibited a positive skewness (Mean Skewness = 0.322). Because standard continuous Gaussian approximations enforce perfect mathematical symmetry (with assumed skewness = 0.000), they fail to capture the inherent asymmetry of the biological data. While combinatorial smoothing rounds out the center of the curve, the extreme right tail remains heavy due to the compounded likelihood ratios of ultra-rare allele combinations. Because forensic operational thresholds are inherently drawn in this extreme tail, the symmetric continuous model leads to a faster decay than the true biological data. Our simulations demonstrated that at operationally relevant thresholds (>+3σ), the continuous model underestimates the false positive risk with profile deviations exceeding four orders of magnitude.

The FFT-based approach natively preserves these discrete boundaries and asymmetrical geometries, simultaneously correcting for low-locus probabilistic gaps and the severe risk underestimations caused by high-locus tail skew.

### 3.5. Establishing Dynamic log_10_LR Thresholds

To calculate the dynamic threshold for a specific set of loci, the FFT arrays of the set’s loci are multiplied together; an operation that efficiently executes the mathematical convolution of their individual distributions. IFFT is then applied to transform this combined data back into the probability domain. This IFFT reconstructs the discretized aggregate PMF for that unique multi-locus combination. By calculating the right-tail cumulative probability from this reconstructed distribution, the algorithm can instantly pinpoint the ${log}_{10}{LR}_{threshold}$ required to cap the expected adventitious matches at the user-defined operational limit.

**Figure 5:**
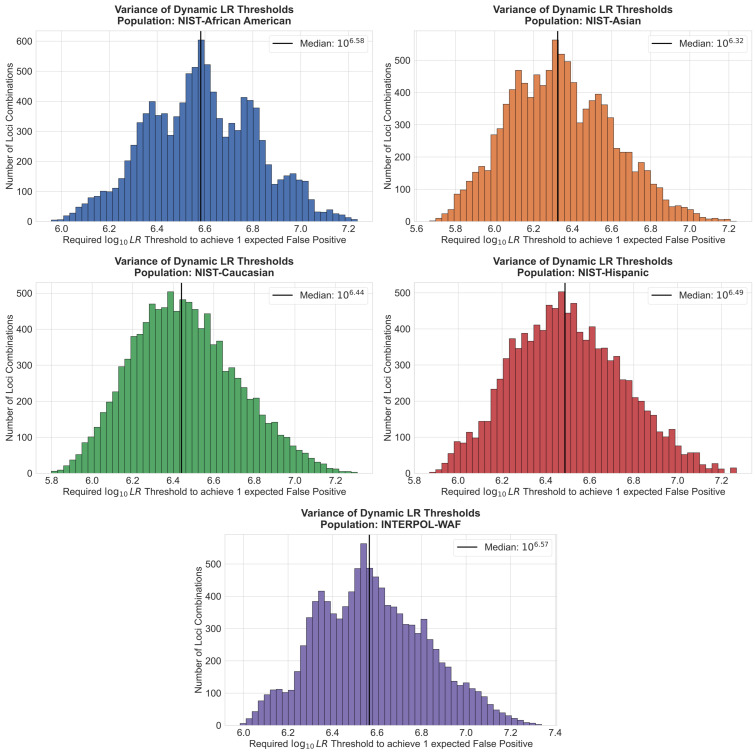
Distributions of ${log}_{10}{LR}_{threshold}$ values required to achieve an expectation of 1 false positive per search for all 10,626 unique sets of 4 loci. Distributions are shown for the 5 reference populations. The black line indicates the median value.

As shown in Supplementary Table S1, extreme locus-to-locus heterogeneity has a direct impact on the diversity of ${log}_{10}{LR}_{threshold}$ values required to maintain a safe false positive rate. Figure 5 shows the analysis of all possible four-locus combinations (_24_C_4_ = 10,626 unique sets of loci). Across the INTERPOL-WAF dataset, the dynamic threshold required to achieve an expectation of one false positive per search ranges from a minimum of 5.98 for the least informative combinations up to 7.34 for combinations containing highly polymorphic markers. Similar values were also seen for the four NIST populations.

**Figure 6:**
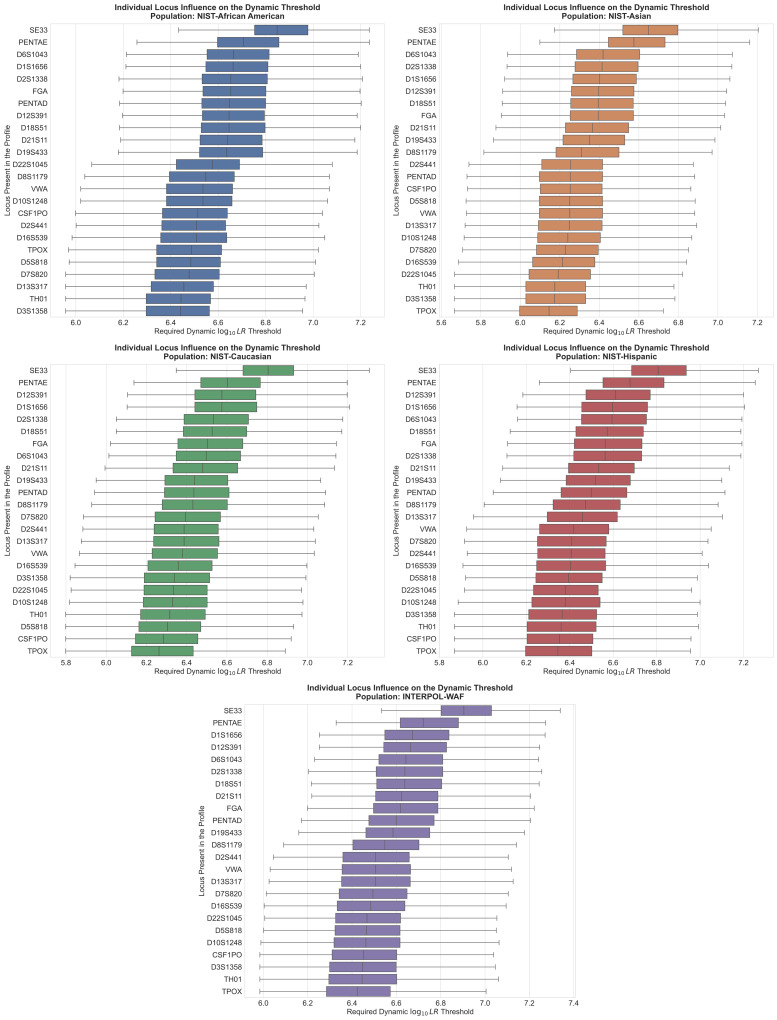
Individual contributions of loci present within the 4-locus matches. The influence of each of the 24 loci is represented for each reference population.

To determine the specific genetic drivers of this variance, the individual contributions of loci present within these four-locus profiles were evaluated (Figure 6).

In all five assessed populations, the presence of universally polymorphic loci, such as SE33 and PENTAE, acted as statistical heavyweights, consistently forcing the required dynamic threshold to be higher due to the rarity of coincidental matches at those markers. Conversely, less informative markers with fewer alleles, such as TPOX and TH01, consistently required lower compensatory thresholds. This analysis revealed that the dynamic algorithm actively compensates for underlying demographic shifts in heterozygosity. While SE33 and PENTAE universally demanded the highest thresholds, the hierarchy of subsequent loci shifted depending on the genetic background of the database. For example, D12S391 emerged as a primary threshold driver within European and Hispanic populations, whereas D6S1043 displaced it within the Asian and African American datasets. Ultimately, this massive spread in required thresholds mathematically demonstrates the inherent danger of applying static thresholds (i.e., locus-count) rather than their specific identities; such thresholds will inevitably either over-penalize strong profiles or fail to adequately account for profiles with lower discrimination power.

### 3.6. Expected Versus Observed False Positives

**Figure 7:**
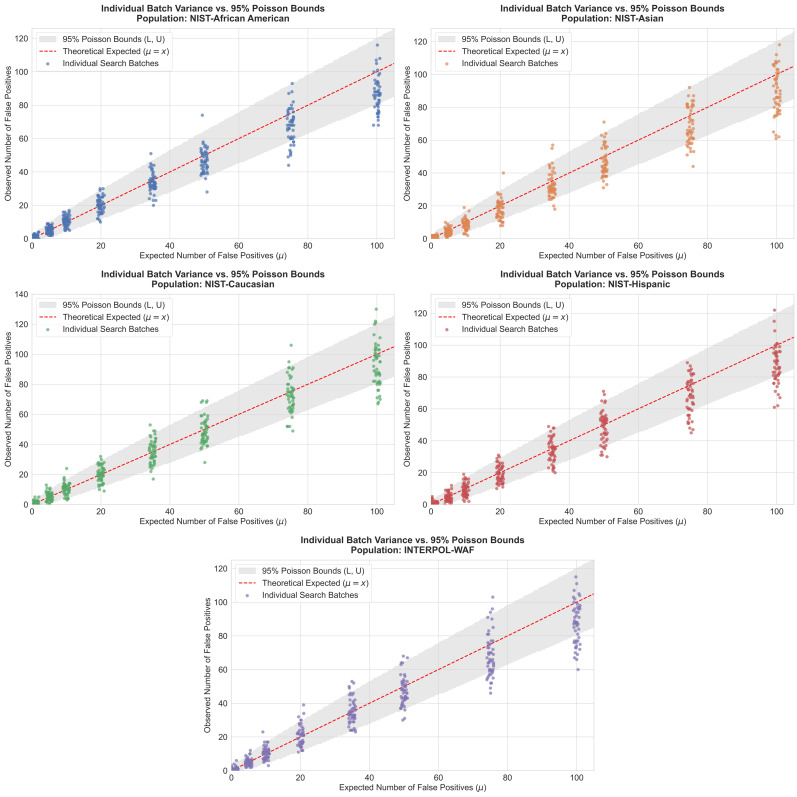
Individual Batch Variance vs. 95% Poisson Distribution Bounds. Each graph corresponds to one of the reference populations. The light gray shaded area represents the 95% Poisson distribution bounds, indicating the expected lower and upper limits for the statistical variance. The red dashed line indicates the theoretically expected number of false positives, marking the point where the observed values match the mathematical expectation (μ = x). The blue dots represent the actual number of observed false positives recorded for each individual search batch during the simulation.

A critical metric for evaluating the reliability of a database searching framework is its ability to accurately predict and control the rate of adventitious matches. Because the dynamic threshold explicitly accounts for the number of pairwise comparisons, it inherently establishes a theoretical, expected number of false positives for a given database size and LR threshold. To rigorously validate this predictive capability, a Monte Carlo simulation was conducted utilizing four-locus matches, representing one of the genetic thresholds where the risk of coincidental matching is most acute. For each of the five reference populations, 1000 simulated stains were searched against a background database of 50,000 individuals across 50 iterations. The framework was programmed to target a specific range of expected false positives (ranging from 1 to 100 per simulation batch). The mathematically expected number of false positives was then compared against the observed number of coincidental matches that successfully cleared the dynamic threshold. The results shown in Figure 7 demonstrate that the dynamic framework is accurately calibrated across all tested genetic backgrounds.

This is because the Monte Carlo simulations model both the query stains and the reference database as originating from a shared subpopulation, thereby mirroring the theoretical assumptions of the FFT algorithm. For operationally relevant risk targets (1, 5, 10, 20, 35 and 50 expected false positives), the mean observed number of false positives demonstrated near-perfect alignment with the mathematically expected targets (the y = x calibration line). For example, across all five reference populations, a strict theoretical target of 10 expected false positives yielded an average of 8.8 to 10.7 actual matches, proving the framework’s precision in simulated database environments.

Furthermore, while verifying the average alignment is essential, the proposed method of probabilistic upper specification relies on the assumption that the observed false positives follow a Poisson distribution. This is reasonable as coincidental matches in a large database search constitute rare, independent events. As illustrated by the scatter plots in Figure 7, the observed false positives for each individual simulation batch were evaluated against the theoretical 95% Poisson confidence intervals (Lower bound L and Upper bound U). Across all tested populations, approximately 95% of the actual false positive counts for individual searches successfully fell above L and below U. This critical observation demonstrates that the framework’s estimates are not only valid on average but also accurately predict the theoretical statistical properties and risk boundaries for individual searches, provided the underlying independence assumptions hold.

Nevertheless, a slight, inherently conservative deviation emerges at the extreme tail of the distribution. At a highly relaxed target of 100 expected false positives, the simulations consistently yielded marginally fewer actual matches than mathematically predicted (averaging between 86.5 and 93.1). This minor shift likely reflects the discrete nature of biological loci and the probabilistic gaps discussed previously. Ultimately, the discrete nature of the loci ensures that the algorithm remains highly accurate at standard operational levels while naturally defaulting to a slightly conservative, protective stance when limits are pushed to their mathematical extremes.

### 3.7. Practical Application of Dynamic LR Thresholds: A Scenario-Based Example

To illustrate the practical implementation and advantages of a dynamic ${LR}_{threshold}$, we simulated four distinct database search scenarios (A through D). These scenarios are presented didactically to demonstrate the underlying mechanics of the framework and how the threshold adapts to varying parameters; they are not intended as an empirical validation of operational performance.

**Figure 8:**
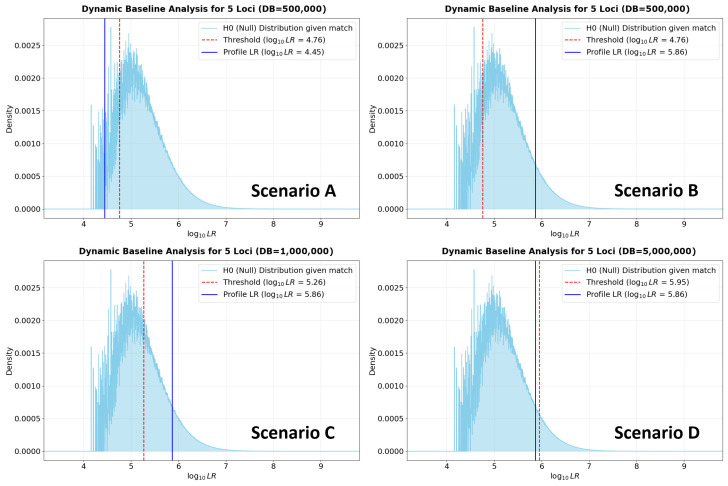
Practical application of the dynamic threshold framework across four simulated database search scenarios. All scenarios evaluate a five-locus match with a strict risk tolerance of one expected false positive per search. The light blue shaded region represents the Multi-Locus Probability Mass Functions (PMFs) of the adventitious match distribution under the defense hypothesis H_0_. The red dashed line indicates the dynamic log_10_LR threshold required to maintain the predefined risk tolerance, while the solid dark blue line represents the calculated log_10_LR of the observed crime scene profile. (**A**) A baseline search against 500,000 individuals using a profile composed of highly common alleles. The profile’s evidential weight falls below the dynamic threshold, correctly resulting in rejection. (**B**) A search against the same 500,000-person database where the profile includes a rare allele. The increased biological weight safely surpasses the threshold, flagging the match for review. (**C**) The database size is doubled to 1,000,000 individuals, inherently raising the dynamic threshold. The rare profile’s weight remains robust enough to exceed this new limit. (**D**) The database is expanded to 5,000,000 individuals. To mitigate the drastically increased risk of coincidental matches across a vast search space, the dynamic threshold shifts significantly upward, resulting in the rejection of the profile.

Across all scenarios, a five-locus match (D10S1248, D12S391, D13S317, D16S539, and TPOX) was evaluated using the INTERPOL-WAF allele frequencies and θ=0.01. The risk tolerance was strictly set to a maximum of one expected false positive per database search. The outcome scenarios are presented in Figure 8.

Scenario A establishes the baseline. The search is conducted against a database of 500,000 simulated unrelated individuals. Based on the multi-locus PMF for these five specific markers, the dynamic threshold to maintain a maximum of one false positive is calculated at a ${log}_{10}{LR}_{threshold}=4.76$. The observed profile consists of highly common alleles (D10S1248: 13,14; D12S391: 18,18; D13S317: 11,12; D16S539: 11,11; TPOX: 8,11), yielding a ${log}_{10}LR=4.45$. Because the evidential value of the profile (4.45) falls below the dynamic threshold (4.76), the match would be rejected, considering that the risk of a coincidental match in a database of this size is too high for such a common profile.

Scenario B demonstrates the system’s sensitivity to informational weight rather than mere locus count. The database size remains 500,000 (meaning the ${log}_{10}{LR}_{threshold}$ remains 4.76), but the match is altered to include a rare allele at the D10S1248 locus (genotype 8,14 instead of 13,14). Because allele 8 has a low population frequency, the profile’s overall ${log}_{10}LR$ increases, safely surpassing the 4.76 threshold. The system flags this match for review.

Scenarios C and D highlight the system’s ability to mathematically scale with database size while evaluating the same rare-allele profile from Scenario B. In Scenario C, the database size is doubled to 1,000,000 individuals. To maintain the standard of only one expected false positive, the dynamic threshold must inherently increase. However, the evidential weight of the profile containing the rare allele remains sufficiently robust to clear this new, higher threshold (${log}_{10}{LR}_{threshold}=5.26$), resulting in the profile to be considered for review. Conversely, in Scenario D, the database size is expanded tenfold to 5,000,000 individuals. The sheer volume of this database exponentially increases the probability of an adventitious hit. Consequently, the dynamic threshold shifts upward significantly to mitigate this risk (${log}_{10}{LR}_{threshold}=5.95$). Against this massive database, the profile’s ${log}_{10}LR$ is no longer powerful enough to rise above the background noise. The match is subsequently rejected.

Together, these scenarios validate the principle of a dynamic thresholding framework. They demonstrate that the admissibility of a DNA match cannot be determined by the number of loci alone, nor can it be decoupled from the size of the database being searched. By adapting to both the specific genetic rarity of the profile and the statistical realities of the database size, this approach has the potential to minimize the reporting of false positives without discarding statistically powerful, low-locus-count matches.

### 3.8. Empirical Validation Using Simulated Degraded Profiles

**Figure 9:**
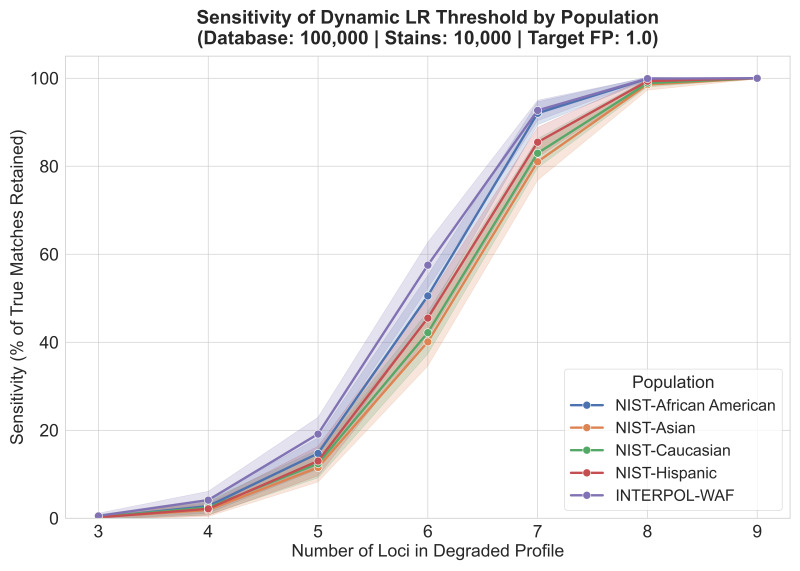
The graph displays the proportion of true matches retained by the proposed dynamic threshold framework as a function of the number of matching loci (from 3 to 9) for simulated searches. Calculations were performed using a user-defined target expectation of false positives μ of 1 and a search space size (T, total number of pairwise comparisons) of 1 billion (10,000 degraded stains queried against a database of 100,000 reference profiles), translating to a per-comparison false positive probability α of 1 in 1 billion. To assess framework reliability and variability, the simulations were divided into 100 equal batches. Solid lines represent the mean sensitivity across batches for each of the five population groups, while the shaded regions indicate ±1 standard deviation, illustrating the consistency of the thresholding approach.

To evaluate the operational efficacy of the dynamic framework, its sensitivity (the proportion of true matches retained) and specificity (the rejection of adventitious matches) were assessed across profiles exhibiting varying degrees of degradation (ranging from 3 to 9 loci). To ensure a biologically realistic test environment, the reference database (*N* = 100,000) and the query stains (*N* = 10,000) were generated utilizing a shared Dirichlet subpopulation model (θ= 0.01). The framework was tasked with maintaining a strict operational risk limit of one expected false positive across each entire batch of one billion pairwise comparisons. As presented in Figure 9 and Supplementary Tables S5–S7, the inclusion of realistic subpopulation clustering demonstrated the severe baseline risk inherent in searching highly degraded profiles. Prior to the application of the dynamic threshold, three-locus and four-locus queries generated an overwhelming volume of adventitious matches across all reference populations. For instance, within the Asian dataset, the three-locus queries generated nearly half a million raw coincidental matches (439,762). However, the dynamic thresholding algorithm successfully neutralized this noise; across all five tested populations and all loci counts, the framework rigidly clamped the number of reported false positives to the designated operational target (averaging between 0 and 4 passed false positives per batch). This absolute control over the FPR naturally dictates the framework’s sensitivity. For severely degraded three-locus and four-locus stains, the algorithm mathematically recognizes the high rate of false positives and compensates by erecting a highly stringent threshold. Consequently, while the noise is successfully blocked, sensitivity is correspondingly suppressed, with less than 1% of true matches passing at three loci, and only 1.87% to 4.13% possessing enough informative power to cross the threshold at four loci. Importantly, the algorithm’s dynamic nature allows it to automatically relax this stringency as the informational content of the profile increases. At five loci, sensitivity across the populations rises steadily (11.54% to 19.14%), and by six loci, the raw coincidental noise drops swiftly, allowing the algorithm to safely retain 40.09% to 57.51% of true matches. By seven loci, the dynamic framework begins to achieve high operational efficiency (81.01% to 92.68%), and at eight and nine loci, sensitivity effectively reaches 100% while successfully discarding all adventitious noise. Within the parameters of this simulation, the dynamic algorithm improves investigative yield for partial profiles, maximizing true positive retention without ever violating the laboratory’s pre-defined risk boundaries.

### 3.9. Comparative Efficiency of Match Filtering

To contextualize the efficiency of the dynamic LR framework against conventional forensic reporting standards, an ROC analysis was performed. To generate the underlying data, a highly degraded casework environment was simulated. A reference database of 100,000 profiles was computationally generated, alongside sets of 1000 random crime scene stains for each locus count ranging from highly degraded three loci) to moderately degraded (seven loci). These stains were cross-searched against the database to generate discrete populations of true matches (true positives) and adventitious coincidental matches (false positives).

The resulting matches were evaluated across three distinct filtering methodologies:

• Method A: A conventional static locus-count threshold.
• Method B: A conventional static likelihood ratio threshold.
• Method C: The proposed dynamic LR framework.

**Figure 10:**
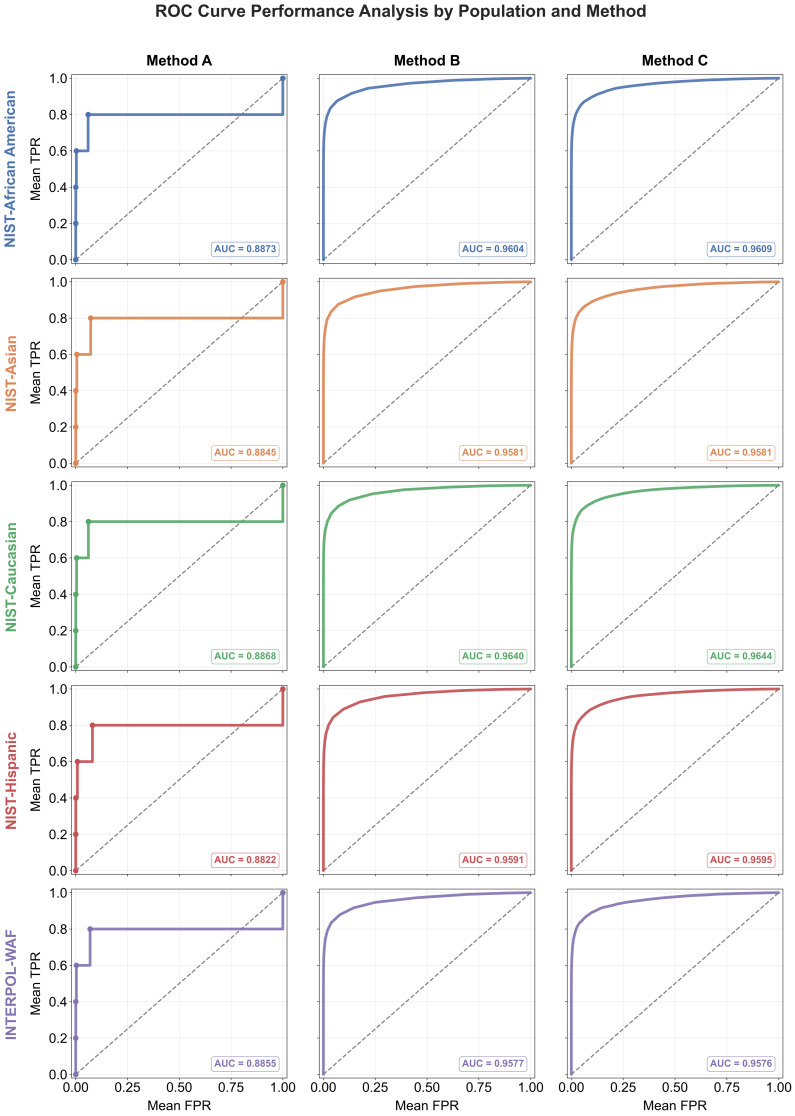
Receiver Operating Characteristic (ROC) curves comparing three DNA match filtering methodologies for degraded profiles (3 to 7 loci) across five reference populations. The grid layout displays the true positive rates and false positive rates independently for each population dataset, simulated using 10,000 crime scene stains cross-searched against a 100,000-profile database. Method (**A**) relies on a conventional static locus-count threshold. Method (**B**) applies a static LR threshold. Method (**C**) represents the proposed dynamic LR framework. Area Under the Curve (AUC) values are provided for each specific method.

To ensure the results were transparent and universally applicable, the entire simulation was executed independently across the five curated population frequency datasets. The false positive rates (FPRs) and true positive rates (TPRs) were evaluated and plotted individually for each population. This generated population-specific ROC curves, allowing for a side-by-side comparison of the three filtering methodologies, as shown in Figure 10.

The conventional approach of relying purely on a hard-coded number of matching loci (Method A) proved to be the least efficient method (Mean Area Under the Curve (AUC) = 0.887). The ROC curve for this method exhibits a distinct, jagged “staircase” trajectory, representative of its discrete nature. Because locus-counting cannot evaluate the genetic rarity of the specific alleles present, relaxing the threshold from six loci to five loci results in a massive horizontal shift along the X-axis representing an influx of adventitious matches without a proportional gain in true positive sensitivity. It indiscriminately discards highly probative five-locus matches composed of rare alleles while accepting biologically common, high-risk six-locus matches.

Transitioning to a static likelihood ratio threshold (Method B) produced a dramatic increase in filtering efficiency (Mean AUC = 0.9659). By evaluating the mathematical rarity of the matching alleles, Method B successfully distinguishes between statistically powerful low-locus profiles and weak high-locus profiles, resulting in a smooth, optimized curve.

The proposed dynamic LR framework (Method C) behaves very similarly to Method B in terms of pure discriminative power (Mean AUC = 0.9664), as both fundamentally rely on the likelihood ratio to rank matches.

While ROC analysis effectively visualizes the discriminative power between true and false matches, it evaluates algorithms on a strictly one-to-one basis. This fundamental limitation masks the severe operational burden caused by massive database searches. An algorithm may possess a strong AUC in a controlled one-to-one test, but a real-world database performs millions of comparisons simultaneously. When searching a database of millions of individuals, even a small FPR will mathematically compound. If these false leads must be manually reviewed by custodians, the algorithm represents an operational failure, regardless of its ROC performance.

To translate the theoretical efficiency into a practical demonstration, the simulation data was subjected to Poisson distribution modeling. Utilizing the baseline coincidental match rates observed during the 1 billion pairwise comparison simulations, the expected number of false positives μ was scaled linearly to represent various current world database sizes. The Poisson probability formula was then applied to calculate the risk of encountering at least one adventitious match during a single casework search. The results, averaged across the five populations, are presented in Table 2.

The results expose the structural weakness of Method A. Decades ago, when forensic databases routinely contained fewer than 100,000 profiles, a static “6-locus rule” was highly safe, carrying a negligible 1.15% risk of triggering a false positive. However, as national databases scale to millions of profiles or interconnect into massive international networks containing tens of millions of records, this static rule becomes statistically untenable. Should the cumulative number of searchable DNA profiles within the Prüm network eventually reach 50 million, applying this exact same threshold carries a 99.70% probability of triggering an adventitious match. This mathematical reality perfectly mirrors operational reports from national database custodians, who note that at the 6-locus level, there is a chance of “more than 60% that it is a false positive” [35]. If a laboratory attempts to recover lost sensitivity by dropping the threshold to five loci, the probability of an adventitious match exceeds 84.92% across all demographics in databases as small as 1 million profiles.

More importantly, this table fundamentally undermines the viability of Method B (fixed LR). While a static LR threshold evaluates evidence better than loci-counting, it remains completely blind to the search space. A fixed LR of 10,000 might successfully suppress noise in a 100,000-person database, but against 50 million profiles, the sheer volume of comparisons will inevitably surface coincidental profiles that exceed that static mathematical boundary. A laboratory utilizing Method B is forced into a continuous cycle of manually escalating its fixed LR threshold as its database grows, arbitrarily sacrificing sensitivity without a mathematically principled foundation.

While Methods B and C perform similarly in ranking the evidential weight of an individual match (as evidenced by their nearly identical AUC scores), Method C possesses a critical advantage that addresses the structural scalability issues inherent in traditional filtering methodologies. By defining the admissibility threshold as a probabilistic upper bound on false positives, or an expected false positive limit rather than a static metric, Method C automatically decouples the laboratory’s error rate from the size of the database. Whether searching a local index of ten thousand profiles or a massive Prüm network of fifty million, the operational risk of an adventitious match is mathematically locked at a constant, laboratory-defined maximum.

Method C absorbs the penalty of database expansion, not by artificially inflating the FPR (like Method A), nor by requiring constant manual recalibration (like Method B), but by dynamically modulating the required LR based on the specific loci tested and the exact size of the search space. Consequently, laboratories can maintain strict operational safety and reduce the manual burden on database custodians without artificially sacrificing the investigative potential of degraded evidence.

### 3.10. Influence of Biological Relatives on the Dynamic LR Threshold

Relatives pose a unique statistical challenge because they share IBD alleles, which gives them an increased probability of sharing genetic markers compared to the general population. Because of this, the match probabilities between relatives are much higher than the random match probability of unrelated strangers [36]. To put this into perspective, the ENFSI guidelines note that while two unrelated individuals might have an approximate random match probability of 1 in a trillion, a parent and child have an approximate match probability of 1 in 100 million, and full siblings share an approximate match probability of 1 in 100,000 [37]. Consequently, close relatives are the main contributors to genetic substructure within DNA databases. Furthermore, the impact of these relatives on the expected number of adventitious matches will only continue to grow over time as more related individuals are systematically added to national databases. When investigators search partial crime scene stains (e.g., three, four, or five loci) or review DNA database matches involving a small number of matching loci, this biological similarity becomes highly problematic. Attempting to match crime scene profiles to a forensic database of any reasonable size based solely on the degree of allele sharing results in numerous false matches between common alleles. Siblings represent a particularly complex threat, as their allele-matching patterns are not always informative enough to easily distinguish them from true donors or random chance [38]. To quantify this threat and evaluate the protective effect of the Dynamic LR strategy, a simulated DNA database of 100,000 reference profiles alongside a separate set of 10,000 crime scene stains were constructed. To accurately model the familial threat, a true parent and a true full sibling were generated for each of the 10,000 simulated stain donors using established Mendelian inheritance laws and IBD probabilities. Because the dynamic LR threshold is continuously calibrated to limit the expected number of unrelated false positives in a 100,000-profile database to exactly 1, this methodology allowed us to precisely measure how often a familial match inadvertently possesses enough statistical weight to breach a barrier designed to stop unrelated strangers.

The results of the simulation, detailed in Table 3, demonstrate the raw vulnerability of relying solely on allele sharing. When searching 10,000 partial stains against a 100,000-profile database (yielding one billion pairwise comparisons) without any mathematical threshold, the high volume of comparisons generated a massive number of adventitious matches. At just three loci, the simulation identified an average of 237,882 raw perfect matches for unrelated strangers. Even at four loci, the database produced over 15,600 unrelated false leads. However, regardless of the initial volume of raw matches, the dynamically calibrated threshold successfully silenced the unrelated FPR to an average of one across all tested loci.

A critical secondary benefit of the dynamic LR threshold is the collateral protection it provides against familial false positives. Because the threshold is established so high as to block the massive volume of unrelated strangers, most matching relatives simply lack the statistical weight to clear it. For parent/child relationships, this protection is nearly absolute. Despite generating an average of 61.9 raw perfect matches at three loci and 11.7 at four loci, the threshold successfully blocked 100% of these false leads, resulting in an average of 0 reported matches across the tested loci combinations. Siblings pose the most complex challenge due to their high degree of allele sharing. At the lowest locus counts (three and four loci), where sibling false positives are highly prevalent, the dynamic LR acted as a highly effective shield. At three loci, the algorithm reduced the 457.4 raw sibling matches down to just 0.8 reported matches (a >99.8% reduction). At four loci, it safely reduced 167.6 raw matches down to just 3.6 reported matches (a >97% reduction). The raw volume of sibling matches drops significantly (falling to an average of 23 raw matches at six loci). However, a higher proportion of these matches begins to pass the threshold, with an average of 9.1 reported matches at six loci. A six-locus match between full siblings possesses genuine statistical power. The dynamic LR framework correctly scores it as strong evidence, reflecting the fact that full siblings cannot be easily distinguished from the true donor without testing additional loci. Importantly, the absolute volume of these reported familial leads remains remarkably low (averaging less than 10 cases per 10,000 searches), making them a highly manageable artifact during post-match investigative triage. Ultimately, this highlights a profound operational advantage over current protocols. Under a standard “6-locus minimum” policy, every single six-locus match would be automatically reported and require manual review by an investigator, regardless of how common or genetically weak the alleles might be. In contrast, the dynamic LR strategy inherently recognizes that not all six-locus profiles are of equal strength. As seen in the data, it successfully filters out a significant portion of six-locus matches (e.g., discarding over 98% of the raw unrelated six-locus matches and over 60% of the raw six-locus sibling matches) simply because their statistical power is insufficient to clear the targeted threshold. By discarding these mathematically weak matches, the system drastically reduces the investigative burden, ensuring that only matches with genuine, undeniable evidentiary weight ever reach an investigator’s desk.

## 4. Discussion

In this study, we demonstrate the advantages of using a dynamic LR framework, based on case risk assessment and specific to the set of matching loci, to facilitate the decision-making process of DNA database custodians and forensic laboratories. This automated framework can include the reviewing of all matches, including the ones that could only be found through keyboard searches, without affecting the level of sensitivity currently accepted by contemporary methods.

Currently, DNA database comparisons are carried out to detect potential matches based on identical allelic values, sometimes allowing one deviation (wildcard or mismatch). To avoid the reporting of too many false positive matches, especially for matches involving a reduced number of shared loci between the DNA profiles, a minimal threshold of a predetermined number of loci is set in nearly all DNA matching software.

In addition to strict matching rules, laboratory-reporting thresholds are often set at static likelihood ratio (LR) values (such as 1000, 10,000, or even 1,000,000) that are frequently described as arbitrary. McCarthy-Allen et al. highlight that these high thresholds are often used to avoid reporting “overstated” low LRs, but they simultaneously waste relevant evidential value [39]. Because overlaps exist between LR distributions for three, four, five, and six-locus matches, the fact that a set number of shared loci acts as a unique criterion reinforces its arbitrary nature. Consequently, potential DNA matches containing a number of shared loci below the minimum requirement are automatically rejected, despite some of these matches having a high likelihood ratio and potential investigative benefit.

Although this simple static strategy maximizes initial specificity—thereby reducing the automated reporting workload to a minimum—it creates significant operational hurdles elsewhere. To recover the high-value matches that the automated system blindly rejects, laboratories must perform “keyboard searches.” Most of these low-locus, high-value matches can only be found through this method, which requires an individualized, manual workflow and specific administrative authorization for the searches to be carried out.

Furthermore, our empirical comparison against continuous baseline models confirms that the discrete nature of DNA match probabilities cannot be safely approximated by standard continuous curves. When encountering probabilities within the distribution and especially at the extreme right-tail, continuous models structurally fail to capture the true risk at operational reporting thresholds. The dynamic FFT framework resolves this by remaining strictly anchored to the discrete combinatorial boundaries of genetic data.

The use of the minimum number of loci as the sole criterion can also affect the efficiency of DNA database searches involving matches of six loci or more. Considering the high volume of profiles that are compared daily in the Prüm DNA exchange network, some experts and stakeholders consider the si (and even seven) loci matching rule highlights ethical concerns due to the high frequency of false positive matches that are reported [35,40,41]. Indeed, previous studies estimated the rate of false positive matches to reach around 60% for six-locus matches but only 6% for seven-locus matches [42,43]. Based on the results presented in Table 2, these numbers are indeed predicted using the Poisson probability formula in a database of 10 million DNA profiles.

Unlike a static threshold, which remains constant irrespective of the database size or the accepted workload required to review the matches, the dynamic nature of our strategy is adaptable and allows for flexibility and responsiveness to different configurations, including the stringency level of the database search and the size of the DNA database. The stringency level of the database search has a direct impact on the size of the list of candidate matches (mainly or entirely composed of false positive matches) that police forces deem manageable to review, since they must verify or disprove each false positive match. In particularly serious criminal cases, the objective may be to review any potential lead, even though the number of matches to review may be extremely high. However, in a less serious criminal case, the objective may be to only review matches that have reached extremely high stringency, thereby reducing the number of false-positive matches, if any. By framing the search criteria around a strict confidence upper bound $\left(U_{95}\right)$, database custodians now have a tool to control their investigative workload that will not exceed their administrative capacity, transforming database searching from a stochastic risk into a predictable, managed process. Several levels of accepted risks, applied differently for high-profile cases or routine daily searches, may be decided by the DNA database custodian as a general principle.

The size of the database is also an important criterion. In 2019, the INTERPOL DNA Unit published the INTERPOL Global DNA Survey, which describes the use of DNA profiling and DNA databases in its member countries [2]. Out of 194 (at the time of the survey publication) INTERPOL member countries, 70 countries reported having a national DNA database (i.e., a searchable repository) with database sizes ranging from several thousand to several million. Our method is applicable to all national DNA databases worldwide, regardless of their current size or future growth. Before applying this framework to national DNA databases, DNA database custodians should consider four parameters: the minimum input requirement, the matching requirement, the associated workload, and the quality assurance standards.

Although the method would allow the reporting of potential matches with a minimum of three shared and matching loci, DNA profiles could only be searched in the first place, provided that they also meet a minimum input requirement. Input requirements were set in the first place to avoid populating DNA databases with very partial DNA profiles, which could easily match a very high number of already stored DNA profiles, thereby leading to a very high number of false positive matches, which would in turn require substantial review time. Due to historical reasons, the countries that initiated their national DNA database in the late 1990s (such as the United States, the United Kingdom and the Netherlands) are still storing DNA profiles that were produced by the first typing solutions (e.g., SGM with six loci + amelogenin) [37]. To address this, we would recommend adjusting input requirements by only allowing the storage of highly degraded DNA profiles if their RMP reaches a minimum value. A similar approach has been applied since 2016 in NDIS, which authorizes the uploading and searching of a forensic DNA profile with a minimum of eight of the original CODIS Core Loci combined with a match rarity of at least one in ten million (equivalent to a log_10_LR equal to 7) [44]. This update of NDIS operational procedures allows profiles that were never searched before to result in new matches of high quality, although it does not allow matches with a low number of shared loci to be reported, as proposed in our study.

Matching requirements applied in this study present an improvement compared to traditional methods. While theoretical frameworks, such as those discussed by Kruijver et al., suggest that a uniform LR threshold is the most efficient approach for searching heterogeneous databases, this optimality assumes that the LR can be accurately characterized across the entire distribution [45,46]. The study from Kruijver et al. notes that while fixed thresholds are optimal in the Neyman–Pearson sense, they do not allow for control over the Probability of Detection (PoD) on a case-by-case basis. In the field of familial DNA searches and in scenarios with a low number of matching loci (e.g., below 10 loci), fixed thresholds often fail to capture true relatives because these profiles may never reach the high LR values typically required to limit false positives in large databases. By employing a dynamic approach based on the (1-α)-quantile of the risk distribution, this study adopts a profile-centered strategy. This allows for a fixed probability of finding matches even when genetic information is sparse, addressing the “out of control” nature of detection probabilities inherent in fixed-threshold searches for low-locus matches.

The workload is probably the parameter that this study could positively impact the most. Resources are needed to operate a DNA database, since the database operators need to generate a report for every match. In many countries, the matches found in the database are evaluated in the DNA laboratories. DNA experts check the raw data, especially the loci that were typed but not reported (below the analytical threshold, for example). This enables DNA database operators to accept or reject potential matches and to communicate their decision back to law enforcement. Police investigators then re-evaluate the match in light of all available case-specific evidence, including additional biometric data, to determine its investigative relevance. At the end of the process, judges again reevaluate the DNA evidence, considering the whole case information to decide whether the DNA profile originated from the person of interest or not. Reducing the risk of reporting a false investigation lead is therefore essential, not only for the police forces that will have to handle each false investigation lead but also for the DNA database operators, the DNA laboratory, and sometimes the judge. Reporting matches that meet a constant measured risk using a dynamic threshold would impact on the whole chain of custody and also eliminate the additional burden of requesting and performing manual keyboard searches since high-probability matches would already be identified automatically.

Finally, adopting a dynamic LR strategy may provide a more rigorous and centralized approach to quality assurance, particularly regarding the underlying population data. To ensure consistency across all searches, the system could rely on a standardized, version-controlled reference file (i.e., library) containing the specific allele frequencies used for calculations. Of course, each DNA database or DNA laboratory would have to create its own library, based on the national allele frequencies used. This would ensure that every comparison, regardless of when or where it is performed, is benchmarked against the same validated dataset, preventing metric drift and ensuring reproducibility. Furthermore, this transition would enhance courtroom transparency by enabling forensic experts to present a standardized level of risk associated with a match, rather than requiring judges to interpret complex, abstract LR scores. Explaining the probability of an adventitious match in terms of known risk thresholds may be more intuitive for legal professionals, directly linking the statistical weight of the evidence to the practical reliability of the identification. The use of FFT offers the ability to calculate a risk based on the exact set of shared loci between the DNA profiles involved in each match. Although the FFT/convolution/iFFT process takes a few milliseconds, it could be envisaged to store a list of pre-calculated sets in an additional library to speed up the process even more. Interestingly, insights from national DNA database custodians confirmed that the number of unique combinations of loci sets found in their database approximated several thousand combinations, significantly smaller than the 16,776,915 unique sets of loci possible when considering matches ranging from 3 to 24 loci. This convergence of loci sets can be attributed to three main factors: (1) kit standardization (national DNA databases and international exchanges predominantly utilize standardized STR kits, leading to a high frequency of overlap at core CODIS, European Standard Set of loci or INTERPOL Standard Set of loci), (2) differential degradation (environmental factors often affect larger STR markers more severely than smaller ones; consequently, many partial matches share the most robust short loci), and (3) country-specific multiplexes (similarities in the forensic infrastructure of contributing countries result in repetitive patterns of shared genetic data across the database search results).

The implementation of the potential reviewing/reporting of a match with fewer than six loci may introduce a necessary trade-off in result interpretation to consider that the match may be highlighting a biological relationship between two related individuals (parent–child or full-siblings). Our data shows that the risk of reporting biological relatives is quite controlled from 3 to 5 locus matches, since their statistical power is often insufficient to clear the targeted threshold. To mitigate the risks associated with this expanded sensitivity, we propose that any lead generated in the 3–5 loci range should be flagged as a “Potential Kinship/Identity Match”, prompting investigators to consider familial origins. Additionally, utilizing an increased subpopulation correction of θ, such as 0.03 suggested by Tièche et al., would ensure that even if a match is adventitious, the weight of evidence is not overstated in the lower range where Maximum Likelihood Estimation models are most volatile [39,47].

It is important to emphasize that this study represents a mathematical proof-of-concept. The validations performed herein utilize synthetic data generated under the same subpopulation models (Balding–Nichols) and independence assumptions inherent to the dynamic framework itself. Consequently, while these simulations demonstrate the internal mathematical consistency of the FFT approach, they do not fully capture the complexities of real-world operational databases. In practice, database searches encounter hidden relatedness and varying ancestry. While these do not affect the validity of the expected value, they will affect the independence assumption required by the Poisson approximation.

Furthermore, our simulated degradation model (random locus dropout) serves as a baseline proxy but does not reflect the structured missingness seen in true forensic casework, which is strongly correlated with amplicon size, kit design, and template quantity. Future validation on live, operational DNA databases of various sizes and across different regions of the world will be necessary to establish external operational validity and assess the framework’s robustness against real-world violations of these underlying assumptions.

## 5. Conclusions

The transition from static locus-count criteria to a dynamically calibrated likelihood ratio framework represents a practical computational improvement in the management of expanding forensic DNA databases. As demonstrated by our study, traditional static rules fail to account for the multiplicity effect; as databases grow to millions of profiles, the sheer volume of pairwise comparisons mathematically compounds microscopic error rates into an unmanageable burden of adventitious matches. By employing a Fast Fourier Transform algorithm, the proposed framework derives match-specific probability distributions and calibrates the reporting threshold based on a user-defined expected false positive limit. This approach effectively uncouples the laboratory’s operational risk from the size of the database, minimizing the incidence of unrelated coincidental matches even at highly degraded three- or four-locus levels. While the current study focuses on improving operational efficiency and courtroom transparency for single-source casework, the underlying concept of a search-corrected risk level provides a foundation for future research in broader forensic applications. Specifically, future studies should investigate whether this dynamic thresholding can be adapted for kinship investigations, where missing data, allele sharing, and complex competing hypotheses routinely obscure true evidential weight. If empirically validated in these complex domains, a risk-based threshold might eventually assist practitioners in evaluating investigative leads while maintaining mathematical control over false positive rates. Ultimately, provided it is successfully validated on real operational data, adopting this scalable framework could allow database custodians to safely evaluate rare, highly informative degraded profiles as global DNA databases continue to expand. To translate this mathematical proof-of-concept into routine practice, these simulated findings must now be confirmed through rigorous empirical testing across national DNA databases of varying sizes and demographic compositions.

## Figures and Tables

**Table 1:** Relationship between the expected number of false positives μ and the operational upper bounds at 95% (U_95_) and 99% (U_99_). The discrete upper bounds were derived using the Poisson cumulative distribution function. Specifically, U_95_ and U_99_ represent the smallest integer k for which the cumulative probability of observing k or fewer false positives is ≥0.95 and ≥0.99, respectively, given the expected mean μ.

Expected Number of False Positives (μ)	95% Upper Bound ($U_{95}$)	99% Upper Bound ($U_{99}$)
0.01	0	0
0.05	0	1
0.1	1	1
1	3	4
5	9	11
10	15	18
20	27	31
50	62	67
100	117	124

**Table 2:** Average Risk of observing 1 False Positive per Single Search according to Database Sizes. The table displays the average Poisson probability of encountering at least one adventitious match during a single casework search. Probabilities were extrapolated from baseline coincidental match rates generated via large-scale computational simulations and averaged across all five population groups.

Database Size	4 Loci	5 Loci	6 Loci	7 Loci
100,000	92.56%	17.24%	1.15%	0.06%
500,000	>99.9%	61.17%	5.64%	0.30%
1 million	>99.9%	84.92%	10.95%	0.60%
5 million	>99.9%	>99.9%	44.01%	2.96%
10 million	>99.9%	>99.9%	68.65%	5.82%
50 million	>99.9%	>99.9%	99.70%	25.92%

**Table 3:** Impact of a Dynamic LR Threshold on adventitious matches with unrelated individuals or biological relatives. This table reports the number of detected matches and reviewed matches (with a log_10_LR above the dynamic log_10_LR threshold) from simulated data (10,000 stains searched against a 100,000-profile database, with the injection of 10,000 parent/child and 10,000 full siblings generated from each of the 10,000 simulated stain donors using established Mendelian inheritance laws and Identity by Descent probabilities).

Biological Relationship	Number of Matching Loci	Number of Detected Matches	Number of Reviewed Matches
Unrelated	3	237,882.0 ± 32,500.9	1.1 ± 1.5
	4	15,669.9 ± 3556.1	0.6 ± 0.7
	5	944.5 ± 262.4	1.2 ± 1.0
	6	59.4 ± 20.2	1.1 ± 1.1
Parent/child	3	61.9 ± 10.6	0.0 ± 0.0
	4	11.7 ± 3.7	0.0 ± 0.0
	5	1.6 ± 1.1	0.1 ± 0.3
	6	0.4 ± 0.6	0.1 ± 0.3
Full-Sibling	3	457.4 ± 25.1	0.8 ± 0.8
	4	167.6 ± 14.0	3.6 ± 1.5
	5	59.2 ± 8.6	7.0 ± 2.2
	6	23.0 ± 4.7	9.1 ± 3.1

## Data Availability

The original contributions presented in the study are included in the article/Supplementary Material, further inquiries can be directed to the corresponding authors.

## Supplementary Materials

The following supporting information can be downloaded at: https://www.mdpi.com/article/10.3390/genes17050499/s1, Figure S1: Progression of the cumulative multi-locus null distributions from 1 to 24 loci; Table S1: Summary statistics of the five reference populations used in this study; Table S2: Synoptic table of all simulations in this manuscript; Table S3: Percentage of Overlap Between RMP Distributions (3 to 8 Loci) by Population; Table S4: Distributional Skewness and Continuous Model Risk Underestimation Across 10,000 Simulated 6-Locus Profiles. Table S5: Raw False Positives Generated (Before Threshold); Table S6: False Positives Passing the Dynamic LR Threshold; Table S7: True Matches Retained/Sensitivity (%).

## Abbreviations

The following abbreviations are used in this manuscript:

AUC	Areas Under the Curve
CDF	Cumulative Distribution Function
CODIS	Combined DNA Index System
ENFSI	European Network of Forensic Science Institutes
FFT	Fast Fourier Transform
FP	False Positive
FPR	False Positive Rate
HWE	Hardy–Weinberg Equilibrium
IBD	Identity by Descent
IFFT	Inverse Fast Fourier Transform
INTERPOL-WAF	INTERPOL Worldwide Allele Frequencies
LR	Likelihood Ratio
NDIS	National DNA Index System
NIST	National Institute of Standards and Technology
Norm W-Dis	Normalized Wasserstein Distance
PMF	Probability Mass Function
PoD	Probability of Detection
RMP	Random Match Probabilities
ROC	Receiver Operating Characteristic
STR	Short Tandem Repeats
STD	Standard Deviation
SWGDAM	Scientific Working Group on DNA Analysis Methods
TP	True Positive
TPR	True Positive Rate
Uβ	Upper bound at a chosen confidence level β
