# Supplementary figures and images for: A Fast-Fourier-Transform-Based Dynamic Likelihood Ratio Framework for Controlling False Positives in DNA Database Matching
Source: Genes (Basel). 2026 Apr 23;17(5):499. doi: 10.3390/genes17050499 (PMC13206489; doi:10.3390/genes17050499)

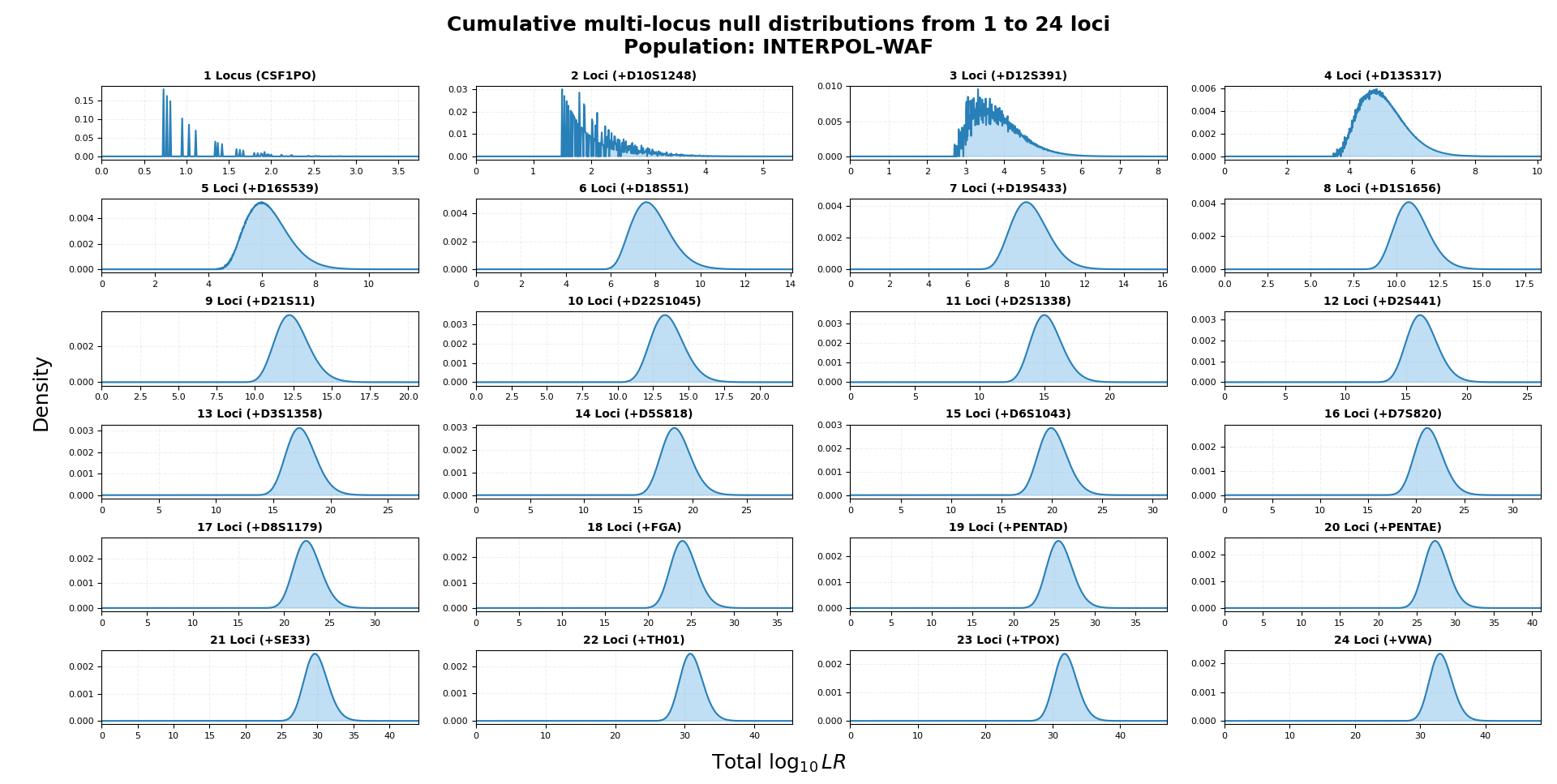

Supplement: Supplementary file 1 [file genes-17-00499-s001.zip › Figure S1.png]
